# Personalizing DNA Cancer Vaccines

**DOI:** 10.3390/jpm15100474

**Published:** 2025-10-02

**Authors:** Annie A. Wu, Kaiqi Peng, Melanie Vukovich, Michelle Zhu, Yuki Lin, Arindam Bagga, TC Wu, Chien-Fu Hung

**Affiliations:** 1 Department of Pathology, Johns Hopkins School of Medicine, Baltimore, MD 21205, USA; awu20@jhmi.edu (A.A.W.); kpeng15@alumni.jh.edu (K.P.); mvukovi1@jh.edu (M.V.); mzhu3605@sas.upenn.edu (M.Z.); ylin155@jh.edu (Y.L.); abagga1@jh.edu (A.B.); wutc@jhmi.edu (T.W.); 2 Department of Oncology, Johns Hopkins School of Medicine, Baltimore, MD 21205, USA; 3 Department of Obstetrics and Gynecology, Johns Hopkins School of Medicine, Baltimore, MD 21287, USA; 4 Department of Molecular Microbiology and Immunology, Bloomberg School of Public Health, Johns Hopkins School of Medicine, Baltimore, MD 21205, USA

**Keywords:** cancer vaccine, DNA, neoantigens, tumor antigens, clinical trial

## Abstract

Recent progress in tumor immunotherapy highlights the important role of the immune system in combating various cancers. Traditionally designed to protect against infectious diseases, vaccines are now being adapted to stimulate immune responses against tumor-specific neoantigens. Both preclinical studies and clinical trials have explored innovative approaches for identifying neoantigens and optimizing vaccine design, advancing the field of personalized oncology. Among these, DNA-based vaccines have become a particularly attractive approach for cancer immunotherapy. This evolution has been driven by improvements in molecular biology techniques, including more precise methods for detecting tumor-specific mutations, computational tools for predicting immunogenic antigens, and novel platforms for delivering nucleic acid vaccines. Personalized DNA vaccines are typically developed through a complex, multi-step process that involves sequencing a patient’s tumor, computational analysis to identify potential targets, and custom vaccine production. In this review, we examine the use of both shared tumor antigens and individualized neoantigens in cancer vaccine development. We outline strategies for neoantigen identification that provide insights into tumor-specific alterations. Furthermore, we highlight recent advances in DNA vaccine technologies, address the current limitations facing cancer vaccines, propose strategies to overcome these challenges, and consider key clinical and technical factors for successful implementation.

## 1. Introduction

Cancer vaccines are gaining increasing attention in oncology due to their ability to activate the immune system to destroy tumor cells without harming normal tissues. Designing such vaccines requires careful selection of target antigens that can trigger precise immune responses [1]. Among these, neoantigens—novel peptides arising from tumor-specific somatic mutations or mutation-independent cancer-specific changes—are particularly promising, as they are not found in healthy cells and are uniquely presented on cancer cells. Neoantigen-based vaccines are often given in combination with immune checkpoint inhibitors (ICIs), a type of immunotherapy that reinvigorates cytotoxic T cells by blocking inhibitory receptors. ICIs have shown notable success in cancers that have a high mutational burden [2,3].

Neoantigens serve as prime candidates for personalized cancer immunotherapy as they are uniquely expressed in each patient’s tumor. Inputting the terms “neoantigens” and “vaccines” into the search engine of clinicaltrials.gov reveals over 100 clinical trials, highlighting the growing interest in this field. While many trials have yet to demonstrate clear therapeutic benefits, some DNA vaccines have shown encouraging results, including extended recurrence-free survival (RFS) [4,5]. These positive outcomes are supported by technological advances in personalized oncology [6]. Notably, next-generation sequencing (NGS) has enabled comprehensive characterization of tumor genomes, providing insights into mutational profiles, transcriptomic changes, and epigenetic alterations. This information facilitates the individualized identification of immunologically relevant targets.

Selecting suitable delivery systems is also essential in vaccine design. Various platforms exist for delivering neoantigens, such as dendritic cell vaccines, tumor cell preparations, viral vectors, peptides, messenger RNA (mRNA), and DNA-based systems. Among these, DNA-based platforms have gained considerable attention. DNA vaccines offer practical benefits, including rapid production, stability, scalability, and strong safety profiles, though improving their delivery efficiency remains a challenge [7].

This review focuses on DNA-based therapeutic cancer vaccines and their role in enhancing antigen presentation and eliciting immunologic responses to tumors. We will discuss first, the classification and use of shared and personalized tumor antigens; second, approaches for identifying highly immunogenic neoantigens for personalized vaccines; third, the range of vaccine delivery platforms with an emphasis on DNA-based technologies; fourth, current challenges and potential strategies to address them; and fifth, clinical considerations for using personalized vaccines in an adjuvant setting in early stage cancers and in the maintenance setting in advanced disease.

## 2. Classes of Tumor Antigens

Tumor antigens can be divided into two categories—shared and personalized—based on how frequently they are expressed among patients [8]. Shared antigens are commonly found in multiple individuals and represent a potential avenue for developing universal, off-the-shelf cancer immunotherapies. In contrast, personalized antigens are unique to an individual’s tumor, offering a more tailored approach that may enhance immune responsiveness. This section outlines the major classes of tumor antigens and reviews their progress in clinical vaccine development.

### 2.1. Shared Antigens

Shared antigens have been extensively studied for their relevance in oncology and include both tumor-associated antigens (TAAs) and tumor-specific antigens (TSAs). TAAs are typically present at elevated levels in cancer cells relative to normal tissue, while TSAs are uniquely expressed by malignant cells [9]. These features make them attractive, though not always ideal, targets for immunotherapy.

TAAs, such as cancer-testis antigens (CTAs), human telomerase reverse transcriptase (hTERT), and lineage-restricted markers like prostate-specific antigen (PSA) or prostate-specific membrane antigen (PSMA), have been widely investigated in vaccine development [10,11,12,13,14,15,16,17,18,19,20,21,22,23,24]. CTAs (e.g., NY-ESO-1, MAGE family proteins) are notable for their limited expression in immunologically privileged sites (ex. testis) and lack of expression in lymphoid tissues, making them potentially highly immunogenic [10]. hTERT is a catalytic subunit of the enzyme telomerase that is upregulated in various tumor types such as glioblastomas, hepatocellular carcinoma, and bladder cancer. Notable examples of vaccines targeting TAAs include the personalized dendritic cell (CD)-based vaccine Sipuleucel-T (Dendreon Corporation, LLC, Seal Beach, CA, USA), which received FDA approval for prostate cancer [25,26,27,28], and options targeting HER2, HER3, or IGFBP2 [29,30,31,32,33]. However, because TAAs are often expressed at low levels in normal tissues raising the risk of autoimmunity, and clinical benefit from single-antigen TAA vaccines has generally been modest [9,34,35].

TSAs may be of viral origin (e.g., HPV E6/E7, EBV, HBV), representing highly specific and immunogenic targets, or non-viral “public” neoantigens, such as recurrent mutations in Kirsten rat sarcoma virus (KRAS), Wilm’s tumor 1 (WT1), or tumor protein p53 [36,37,38,39,40,41,42,43,44,45,46,47]. Vaccines against viral oncoproteins, specifically E6 and E7, are in active clinical development, with multiple candidates advancing for HPV-related cancers [40,41,42,43,44,45,46]. The broader applicability of shared antigens makes them attractive, but their limited specificity and immunogenicity remain major barriers.

### 2.2. Personalized Neoantigens

Personalized cancer vaccines can bolster immune responses against neoantigens that arise from each patient’s distinctive tumor profile. These may be mutation-dependent (e.g., single nucleotide variants, insertions and deletions, structural variants, gene fusions) or mutation-independent, originating from cryptic translation events, splicing, or post-translational modifications [48,49,50,51,52,53,54,55,56,57,58,59,60,61,62,63,64,65,66,67,68,69,70,71,72,73,74,75,76,77,78,79,80]. Single nucleotide variants (SNVs) are the most extensively studied but neoantigens derived from SNVs often resemble normal peptides, limiting their immunogenic potential. In contrast, mutation derived frameshift or fusion neoantigens often display strong immunogenicity while cryptic antigens from the “dark proteome” represent a promising but still underexplored resource in cancer vaccine development [81].

Early approaches using autologous or allogeneic tumor lysates (e.g., M-Vax [AVAX Technologies, Philadelphia, PA, USA] and Melacine [Corixa Corporation, Seattle, WA, USA]) achieved limited success [82,83,84,85]. Advances in next-generation sequencing and bioinformatics now allow systematic identification of high-affinity patient-specific neoantigens, enabling vaccine formulations tailored to individual HLA backgrounds. These strategies aim to overcome tumor heterogeneity and immune escape while maximizing antitumor immunity.

## 3. Identification of Neoantigens

With the increasing need to discover antigens capable of provoking strong immune responses, the identification of novel, clinically relevant neoantigens has become a key objective in advancing personalized cancer vaccine development.

The general workflow for neoantigen discovery consists of three main stages: identifying potential targets, predicting which candidates may be immunogenic, and validating their biological relevance. Once validated, selected neoantigens are incorporated into vaccines using different technological platforms.

To pinpoint relevant neoantigens—whether they originate from genetic mutations or non-mutational mechanisms—the biological process of antigen creation, processing, and presentation should be considered. This cascade includes: (1) DNA transcription into messenger RNA, (2) RNA translation into protein, (3) protein breakdown into peptides by proteasomes, (4) peptide loading onto MHCs, (5) T cell receptor recognition of peptide-MHC complexes, and (6) T cell activation to initiate an immune response [54]. Each of these steps can be modeled or predicted using computational tools, which are critical for narrowing down the vast pool of potential neoantigens to a practical list for experimental validation [54,86].

This section will begin by outlining current strategies for identifying mutation-derived neoantigens, followed by approaches used to detect non-mutational neoantigen candidates. We will then discuss how immunogenicity can be enhanced through predictive modeling and optimization techniques.

### 3.1. Neoantigen Identification and Sources

Identifying genetic alterations in tumors is the initial step in uncovering mutation-based neoantigens. This is commonly achieved using computational tools, including variant calling algorithms, which compare tumor DNA to a reference either through whole genome sequencing (WGS), whole exome sequencing (WES) [87], or targeted sequencing panels. Additionally, RNA sequencing (RNA-seq) can be employed to analyze tumor tissue and matched normal tissues, typically using peripheral blood mononuclear cells (PBMCs) as the normal reference [87,88,89,90,91,92,93,94,95,96,97,98,99]. Variant calling algorithms compare tumor and matched normal DNA, often peripheral blood mononuclear cells, to identify single nucleotide variants (SNVs), insertions and deletions (indels), and structural variants. While multiple variant callers achieve high sensitivity and precision in pure tumor samples [89,90], performance is often compromised by tumor heterogeneity, stromal admixture, and limited sample quality [88]. Formalin-fixed, paraffin-embedded (FFPE) samples, valuable for their ability to maintain tissue architecture and molecular stability, remain widely available for research and clinical use, but fixation introduces chemical alterations to nucleotides that complicate analysis [92,93,94]. Ensemble calling techniques and neural network-based algorithms have helped mitigate these issues [95,96].

RNA sequencing offers both expression profiles and mutation detection, and when aligned wither to the genome or transcriptome, can identify tumor-specific splice variants. Although sensitivity can exceed 85% under optimal conditions, false-positive rates in SNV calling are strongly influenced by read length [97,98]. More recently, single-cell RNA-seq has enabled variant detection at the cellular level, with encouraging sensitivity but ongoing challenges related to dropout effects and low transcript coverage [99]. Large-scale proteogenomic efforts, such as the Clinical Proteomic Tumor Analysis Consortium (CPTAC), have integrated WGS, WES, and proteomic data to connect genetic variants to actual protein products, helping prioritize functionally relevant neoantigens for cancer vaccine development [100].

To uncover tumor neoantigens that do not arise from genetic mutations, it is crucial to apply advanced methods that analyze high-throughput sequencing data. Ribosome profiling (RiboSeq) is a powerful technique that captures fragments of mRNA protected by ribosomes, thereby providing a genome-wide snapshot of active translation in both tumor and normal tissues [101,102]. This allows researchers to examine the actual protein products being synthesized, including those not linked to known mutations. Immunopeptidomics, based on mass spectrometry, directly detects peptides presented by MHC class I on tumor cells [72,103]. Unlike predictive methods that infer potential antigens from DNA or RNA data, immunopeptidomics offers direct evidence of peptide presentation, capturing the antigenic landscape as it exists on the surface of tumor cells. The combined used of RiboSeq and immunopeptidomics enables the identification of novel translation products—particularly those originating from non-canonical or cryptic sources—that may act as immunogenic targets for therapeutic vaccine development.

### 3.2. Antigen Processing, MHC Presentation, and Immunogenicity Prediction

Following antigen creation, peptides must be processed, transported, and loaded onto MHC molecules for recognition by T cells. Determining an individual’s human leukocyte antigen (HLA) genotype is instrumental for neoantigen prediction, since each allele has unique binding preferences. Computational tools such as Opitype, ArcasHLA, ATHLATES, and seq2HLA can infer MHC class I and II alleles from sequencing data, although short-read sequencing often struggles to fully resolve polymorphisms; targeted sequencing remains the gold standard [104,105,106,107,108,109,110].

Over time, numerous computational models have been created for predicting the likelihood of specific peptides to bind to MHC molecules. Earlier algorithms relied on position-specific scoring matrices, whereas more recent models have adopted machine learning approaches such as neural networks trained on large experimental datasets. NetMHC-4.0 and NetMHCpan-4.0 remain widely used for class I binding [111,112,113], while NetMHCIIpan-2.0 and NetMHCIIpan-3.1 provide improved predictions for class II peptides, which are longer and more structurally diverse [114,115,116,117,118]. Incorporation of large-scale peptide elution datasets from mass spectrometry has further improved predictive accuracy [119]. Proprietary platforms such as EDGE and BigMHC integrate deep learning with ligand data to refine prediction across diverse HLA alleles [120,121].

Predicting T cell receptor (TCR) interactions adds another layer of complexity, as recognition depends on peptide sequence, MHC allele, and both TCR α and β chains. Databases including VDJdb, McPAS-TCR, and the Immune Epitope Database (IEDB) [122,123,124,125,126] provide valuable training data, and high-throughput methods such as single-cell TCR sequencing have expanded available datasets [127,128,129]. While benchmarking studies exist [130], most models struggle with generalizability due to the enormous diversity of potential TCR and pMHC pairings [131,132]. Emerging technologies such as single-cell TCR sequencing from platforms like 10× Genomics have the potential to generate expansive, high-throughput datasets, making them promising resources for enhancing future prediction models [133].

Clonality is an important factor in determining the immunogenic potential of neoantigens. Based on their distribution within the tumor, neoantigens can be classified as either clonal or subclonal. Clonal neoantigens are found uniformly across all tumor cells, whereas subclonal neoantigens are restricted to specific subsets of the tumor. Research indicates that clonal neoantigens are more strongly associated with positive responses to immunotherapies, making them attractive targets for vaccine development and other immune-based treatment [134]. However, targeting clonal driver mutations can be challenging, as tumors may evolve mechanisms for evading detection by the immune system, including downregulating the antigen or altering antigen presentation pathways [135,136]. This form of immune escape may explain the limited success of some therapies directed at commonly shared tumor antigens, such as EGFRvIII, which failed to show clinical benefit in late-stage trials [137,138]. Personalized cancer vaccines offer a potential solution by incorporating a diverse repertoire of neoantigens, including both clonal and subclonal targets, to minimize the risk of immune evasion [135]. Numerous computational tools have been developed to estimate clonality using bulk tumor sequencing data [139]. These include FastClone (University of Michigan School of Medicine, Ann Arbor, MI, USA) [140], PyClone (Shah Lab, BC Cancer Agency, Vancouver, BC, Canada) [141], and sciClone (Shattuck Lab, Washington University, St. Louis) [142]. For greater resolution, single-cell sequencing enables the identification of specific tumor cell populations harboring distinct mutations, providing deeper insights into clonal architecture. Additionally, spatial transcriptomics is emerging as a powerful technique to complement single-cell analyses by mapping gene expression in the spatial context of the tumor. Spatial transcriptomics can shed light on the functional relationships between tumor cells and the microenvironment, including immune cell infiltrates. This means spatial transcriptomics provide insight into cell–cell proximity and contact, helping to distinguish between potential and functional interactions. Additionally, it enables the identification of spatial niches within the tumor microenvironment, revealing how cellular composition, density, and localization contribute to interactions between tumor cells and surrounding stromal and immune populations.

### 3.3. Prioritization and Validation of Candidate Neoantigens

With thousands of candidates generated per tumor, ranking and selection are necessary to focus validation efforts. With numerous prediction tools available, each focusing on identifying top-ranking neoantigens based on metrics such as MHC affinity or TCR recognition, a major challenge is how to unify these outputs into a coherent framework for selecting a manageable number of candidates for experimental testing. Open-source pipelines such as pVACtools (Griffith Lab, Washington University School of Medicine, MS, USA) and OpenVax (Icahn School of Medicine at Mount Sinai, New York, NY, USA) integrate outputs from multiple prediction models and apply scoring thresholds to nominate high-confidence epitopes [143,144,145,146]. Still, computational predictions require experimental validation, since many candidates fail to stimulate effective T cell responses.

Validation typically involves patient-derived T cells, obtained from PBMCs or TILs, co-cultured with antigen-presenting cells (APCs) pulsed with candidate peptides. Functional assays, including enzyme-linked immunosorbent spot (ELISpot) and intracellular cytokine staining, assess interferon gamma (IFN-γ) production and T cell activation [147,148,149,150]. Functional killing assays have been developed to assess the cytotoxic ability of T cells [150,151,152]. Expanded approaches such as targeted TCR sequencing, single-cell profiling (e.g., TCR-sqe and scRNA-seq), and the mutation-associated neoantigen functional expansion of specific T cells (MANAFEST) assay allow sensitive detection of neoantigen-reactive T cells, even when rare [153,154,155,156,157].

Preclinical testing in standard and humanized mouse models remains important for assessing in vivo immunogenicity and antitumor efficacy [158,159]. However, resource intensity and limited predictive power constrain their widespread use. Challenges also persist in translating predictions into effective immunity. These include antigens that are poorly cross-presented, unstable, or rapidly degraded; others suppressed within the tumor microenvironment [62,63,160,161,162,163,164]. Naïve T cell priming often depends on higher levels of peptide-MHC complexes than tumors naturally provide, a limitation that can be mitigated through strong adjuvants and well-designed vaccine platforms [165]. While neoantigen peptides are highly promising targets for cancer immunotherapy, comprehensive strategies must be employed to avoid missing potentially effective epitopes.

## 4. DNA-Based Vaccine Platforms

**Figure 1:**
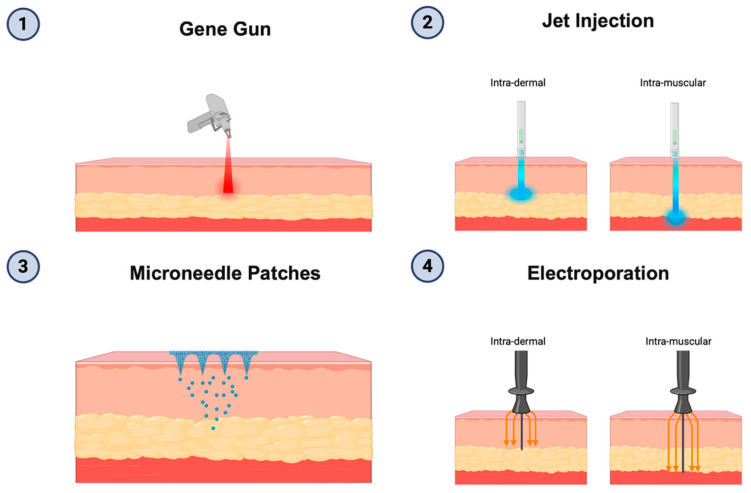
Delivery of DNA vaccines. Outside of conventional intramuscular injection, there are four common strategies used to deliver DNA vaccines. 1. Gene gun, which propels DNA-coated particles into the epidermis. 2. Jet injection, which delivers DNA into the intradermal or intramuscular compartments using high-pressured liquid streams. 3. Microneedle patches, which create microchannels in the skin to release DNA into the dermis. 4. Electroporation, which uses brief electrical pulses that enhances DNA uptake by increasing cell membrane permeability after intradermal or intramuscular injection [4,5]. Created in BioRender. Wu, T. (2025) https://BioRender.com/h276moj (Accessed on 26 July 2025).

DNA vaccines have been used in fewer clinical studies compared to RNA-based platforms. One of the key challenges is that DNA must reach the cell nucleus to be transcribed, requiring it to cross the plasma membrane and the nuclear envelope [166,167]. However, DNA vaccines offer several unique benefits, and improvements in delivery methods could enhance their future application. Figure 1 displays the diverse DNA vaccine delivery strategies and types of DNA-based constructs in current vaccine development.

The DNA vaccine safety profile has been evaluated in multiple clinical trials [168,169,170]. Research has shown that there is an extremely low likelihood of DNA integrating into the host genome, less than that of naturally occurring spontaneous mutations [167,171]. Additionally, DNA vaccines are inherently more stable than RNA, allowing them to be administered in unformulated or “naked” form, which often results in reduced adverse reactions [172,173,174]. RNA vaccines, however, often entail delivery via lipid nanoparticles (LNPs) containing polyethylene glycol (PEG), a component that has been linked to hypersensitivity reactions in some individuals. The ability to administer DNA without LNPs also contributes to lower manufacturing costs and facilitates easier large-scale production [167,175]. Another advantage of DNA vaccines is their prolonged intracellular persistence. Once inside the nucleus, DNA can remain intact and transcriptionally active for several months without integrating into the genome. This contrasts with RNA molecules, which are more transient and rapidly degraded in the cytoplasm [176].

Various strategies have been developed to improve the delivery efficiency of DNA vaccines, as summarized in Figure 1. Because DNA must traverse the cell membrane and nuclear envelope to initiate transcription, intramuscular (I.M.) injection of unformulated DNA often results in poor cellular uptake. To overcome this, electroporation is commonly used, which temporarily increases cell membrane permeability and significantly enhances DNA entry and immune activation. However, the reliance on specialized electroporation devices presents practical challenges for widespread vaccine distribution, as highlighted during the coronavirus disease-2019 (COVID-19) pandemic [177]. Alternative delivery methods are under investigation to broaden the applicability of DNA vaccines. These include physical techniques, such as the gene gun, which propels DNA-coated metal particles into tissue; jet injection, which delivers DNA using a high-velocity fluid stream without needles; and microneedle patches, which use tiny projections to penetrate the skin. These methods are delivered intradermally (I.D.), which has demonstrated improved immune responses compared to traditional I.M. routes. This enhancement is likely due to the fact that the skin has a high density of APCs, enabling more efficient DNA uptake and subsequent antigen presentation [178,179]. Several clinical trials have employed these delivery techniques. In a phase I study (NCT00199849), the gene gun was utilized for the delivery of a DNA vaccine encoding NY-ESO-1 antigen (pPJV7611). Among 15 individuals with no baseline immune response, 33% showed CD8+ T cell responses and 93% exhibited CD4+ T cell responses after vaccination. Despite these promising results, the durability of the immune response was limited, possibly due to suppression by regulatory T cells [13]. Another example is GNOS-PV02 (NCT04251117), a personalized DNA vaccine delivered intradermally followed by electroporation, which led to CD4+ and CD8+ neoantigen-specific T cell responses in 86.4% of patients enrolled in the ongoing phase I/IIa trial [5].

Various alternative strategies have been explored to enhance DNA vaccine immunogenicity by targeting APCs, especially DCs. One approach involves encoding the antigen in fusion with heat shock protein 70 (HSP70), which interacts with specific receptors on DCs, promoting uptake and facilitating cross-presentation [180]. The pNGVL4a-Sig/E7(detox)/HSP70 DNA vaccine, which encodes HPV-16 E7 fused to HSP70, was given to patients with HPV16-positive cervical intraepithelial neoplasia in a phase I/II clinical trial (NCT00121173) [181]. Higher vaccine doses were associated with greater T cell responses. Although not statistically significant, in the highest dose cohort (3 mg), compete histologic regression occurred in 33% (3/9) of participants, slightly higher than the approximately 25% expected in unvaccinated cohorts. Another strategy uses fms-like tyrosine kinase 3 ligand (FLT3L), which binds receptors on DCs stimulating their growth and development. A DNA vaccine, GX-188E, incorporating FLT3L and HPV16/18 E6 and E7 genes, was assessed in a phase II clinical trial (NCT02139267) for HPV-related cervical lesions [182,183]. The trial had promising results. At 20 weeks, histopathologic regression was observed in 52% (33/64) patients, and 73% of those with regression demonstrated HPV clearance. At 36 weeks regression increased to 67% (35/52) with HPV clearance in 77% of regressors. Additionally, fusing target antigens to calreticulin (CRT) can enhance DC-mediated antigen processing. CRT provides a signal to “eat-me”, encouraging DCs to engulf and present the encoded antigens. The pNGVL4a-CRT/E7(detox) vaccine, designed for HPV-positive head and neck carcinomas, was tested in a phase I clinical trial (NCT01493154) [184]. However, the trial was terminated, and no results were posted.

To further increase DNA vaccine stability and delivery efficiency, preventing degradation by nucleases is crucial. Encapsulating DNA within synthetic carriers can improve protection and facilitate cellular uptake. An example is Amolimogene (ZYC101a), which uses poly-lactide-co-glycolide microparticles to deliver a DNA plasmid that encodes HPV-16 and HPV-18 E6/E7 epitopes. This formulation was evaluated for patients with HPV-positive cervical dysplasia in a phase II/III trial (NCT00264732) [185,186]. Results showed that histologic resolution was observed in 43% of vaccinated patients as opposed to 27% of the placebo patients; this difference in resolution was not significant. However, in women under 25 years of age there was a significant difference between treatment arms with vaccinated patients experiencing a resolution of 70% compared to 23% in unvaccinated patients. Furthermore, nuclear localization signals (NLS) can be attached to the DNA vector to enhance transport through the nuclear pore complex into the nucleus, improving transcription efficiency [187,188,189].

Recent advances in DNA vector design have introduced alternative platforms such as doggybone DNA (dbDNA), minicircle DNA (mcDNA), and nanoplasmid DNA, which offer distinct advantages over conventional plasmid DNA (pDNA). Traditional pDNA contains bacterial elements and antibiotic resistance genes, which can induce unwanted immune responses and raise safety concerns due to the potential contribution to antibiotic resistance [190]. In contrast, these newer constructs are engineered to remove such prokaryotic components, reducing the risk of inflammation and improving safety. Moreover, the reduced size of their backbones facilitates more efficient cellular uptake and gene expression [191,192]. Minicircle DNA was first developed in 1997 [193]. It is produced by inserting a plasmid into bacterial cells, where a site-specific recombinase removes the bacterial backbone, leaving only the expression cassette which contains the gene of interest and its regulatory sequences [194,195]. Although mcDNA demonstrates improved transfection performance, its production process is complex and costly, and residual bacterial sequences may still be present after processing. Nanoplasmid DNA, introduced in the early 2000s, addressed some of these challenges by incorporating an antibiotic-free selection mechanism. Plasmids can be selected without antibiotic resistance genes by relying on antisense RNA (RNA-OUT) to silence the expression of a toxic gene (sacB) [196,197]. Although a small portion of the bacterial sequence remains, nanoplasmids have demonstrated significantly enhanced gene delivery, with some studies reporting an increase in transfection efficient up to a tenfold compared to standard pDNA [192,198]. The most recent innovation, dbDNA, was developed in 2017. This approach completely removes the need for bacterial propagation by using a cell-free, enzyme-driven method. Enzymes such as Phi29 DNA polymerase and protelomerase are used to synthesize a covalently closed linear DNA molecule in vitro [199]. This process allows for rapid, large-scale production of simplified DNA constructs, positioning dbDNA as a promising gene therapy vector for vaccine development.

### Personalized DNA Vaccine for Cancer Immunotherapy

**Figure 2:**
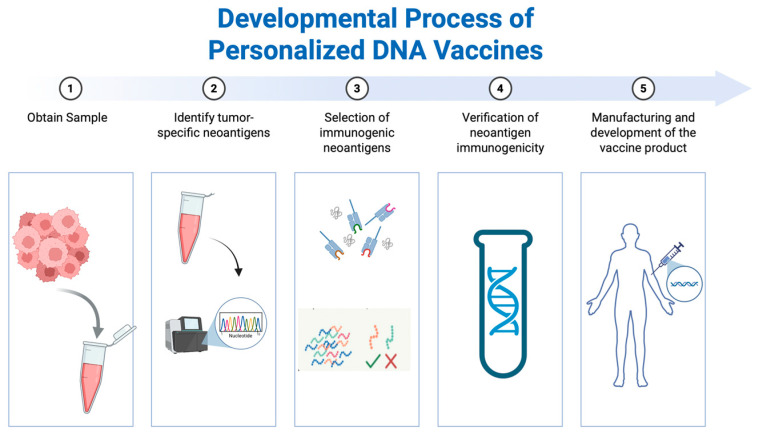
Pipeline for Personalized Cancer Vaccine Development. 1. Obtain Sample. Tumor tissue or blood is collected from the patient with PBMCs or matched normal tissues as germline controls—the source of genetic material. 2. Identify tumor-specific neoantigens. Subsequently, sequencing and bioinformatics analyses are performed to detect somatic mutations and generate a list of potential neoantigen candidates. Mutation-dependent neoantigens can be detected using variant callers while mutation-independent neoantigens are detected using RiboSeq or immunopeptidomics. 3. Selection of immunogenic neoantigens. Computational tools prioritize neoantigen candidates based on predicted MHC binding, TCR recognition, clonality, and dissimilarity to self. 4. Verification of neoantigen immunogenicity After potential neoantigen candidates are identified, possible peptides are experimentally validated for their ability to activate T cells using TCR activation assays or T cell killing assays. 5. Manufacturing and development of the vaccine product. The selected neoantigens are encoded into a DNA vaccine platform and administered back to the patient. Created in BioRender. Wu, T. (2025) https://BioRender.com/uefdnut (Accessed on 26 July 2025).

Recent progress in personalized cancer vaccine development has emphasized identifying individual tumor mutations to design neoantigen-based therapies tailored to each patient. Interestingly, tumors with high TMB are considered more suitable for such approaches due to the increased likelihood of producing immunogenic neoantigens [200,201,202]. The developmental pipeline of personalized cancer vaccines, starting from the acquisition of a sample to the production of the vaccine is summarized (Figure 2). Numerous vaccine platforms are currently advancing through clinical trials, with companies including Moderna, Merck, BioNTech, and Genentech leading late-stage development efforts in phase II and III clinical studies.

Key clinical trials involving DNA vaccines developed for personalized therapies is summarized (Table 1). These vaccines are usually injected via intradermal (I.D.) or intramuscular (I.M.) injection, often with special devices including electroporation or needle-free injection to enhance delivery. Each study varies in vaccine design, combination treatments (like checkpoint inhibitors or cytokines), and route of administration, with DNA vaccines tested in various cancer stages and tumor types. While a few have reported promising initial data, many remain ongoing or have yet to publish clinical results.

One of the earliest trials using DNA vaccines for personalized medicine focused on cancers of the lymphatic system. A phase I clinical study (NCT01209871) published by Thomas et al. in BMC Cancer [212], tested a DNA vaccine designed for patients with untreated asymptomatic lymphoplasmacytic lymphoma (LPL) [212]. This vaccine consisted of a recombinant plasmid DNA encoding a chemokine called MIP3α, which was fused to autologous lymphoma LPL-derived Ig single chain variable fragment (scFv), an endocytic surface receptor on APCs to improve targeting to dendritic cells. It was delivered intradermally using a needle-free injector at three time points (weeks 0, 4, and 8). The trial showed that this approach was capable of stimulating an immune response, providing early evidence that personalized vaccines could work in blood cancers. A follow-up analysis published in Nature Communications confirmed durable immune activation, while also revealing a dichotomous response: the vaccine significantly reduced clonal B-cell subpopulations but had limited impact on plasma cell-like tumor clones, indicating a potential mechanism of immune resistance [213].

Subsequently, DNA vaccines have been tested for solid tumors in clinical trials. A clinical trial (NCT02348320) tested a personalized polyepitope DNA vaccine for triple-negative breast cancer (TNBC), particularly in patients with persistent disease following neoadjuvant chemotherapy [4]. Published by Zhang et al. in Genome Medicine, the study showed that most patients developed T cell responses to one or more of their vaccine-targeted tumor antigens [4]. In a phase I clinical trial, 14/18 vaccinated participants had detectable neoantigen-specific responses and 87.5% of vaccinated patients were recurrence free at 36 months as opposed to 49% of unvaccinated patients. This trial also highlighted the role of computer-based tools in selecting which neoantigens to include, as candidate neoantigens ranked highly by prediction models were more likely to trigger immune responses.

In pancreatic cancer, there has been a phase 1 clinical trial (NCT03122106) testing a personalized neoantigen DNA vaccine for surgically resected pancreatic cancer with adjuvant chemotherapy. There was at least one patient with widely metastatic PDAC who had complete remission following the personalized neoantigen DNA vaccine [214].

Researchers have also explored personalized DNA vaccines in prostate cancers. A trial in prostate cancer (NCT03532217) combined personalized DNA vaccines with a shared prostate cancer vaccine (PROSTVAC) and two checkpoint inhibitors (nivolumab and ipilimumab). According to an abstract by Shah et al., 79% of participants with hormone-sensitive prostate cancer that had metastasized were given the personalized DNA vaccine, and the treatment was well tolerated [205]. Preliminary results showing upregulation of activation, co-stimulatory, and co-inhibitory markers post-treatment suggested immune priming, even in a tumor with low TMB such as prostate cancer.

In liver cancer, the GNOS-PV02 trial (NCT04251117) tested a personalized DNA vaccine for hepatocellular carcinoma (HCC). This vaccine includes up to 40 tumor-specific neoantigens and was given with interleukin-12 (IL-12) to boost T cell activity and pembrolizumab, a PD-1 inhibitor. An early report published in Nature Medicine by Yarchoan et al. showed that these DNA vaccines could activate CD4+ and CD8+ effector T cells in HCC patients [5], and 86.4% of the patients evaluated had neoantigen-specific T cells responses. Clinically, 30.6% (11/36) patients had an objective response, including 8.3% (3/36) of patients with a complete response. A subset of patients had tumor infiltration of T cells. These findings suggest that DNA vaccines could help convert advanced liver tumors, often resistant to immunotherapy due to low T cell infiltration, into more responsive ones.

For skin cancers, EVX-02 is a personalized DNA plasmid that targets neoantigens given with anti-PD1 to patients with complete resection of late stage melanoma in a phase I/II clinical trial (NCT04455503). Results demonstrated increases in T cell responses that were long-lasting, with CD4+ and CD8+ T cells reactive to vaccine antigens [211].

More recent clinical trials are exploring how personalized DNA vaccines might benefit brain cancers. One ongoing study (NCT04015700), nearing completion, is testing a neoantigen DNA vaccine (GNOS-PV01) for newly diagnosed, unmethylated glioblastoma. The personalized DNA vaccine is delivered with IL-12 using an electroporation device. Preliminary results of a subset of patients showed detectable responses, with 6 out of 7 patients with a sustained T cell response against cancer neoantigens after treatment [215].

Efforts are also underway to apply DNA vaccine technology to treat lung cancers. A phase II clinical trial (NCT04397003) tested a neoantigen DNA vaccine with the programmed death-ligand 1 (PD-L1) inhibitor durvalumab for extensive-stage small cell lung cancer (ES-SCLC). Although still in early stages, with just a few patients enrolled so far, the clinical trial is expected to run through 2029. The long timeline reflects the complexity of making personalized vaccines and the need for extended follow-up in aggressive cancers. Taken together, these trials show how DNA vaccines are gradually becoming a key part of personalized cancer treatment.

Combining DNA vaccine therapies with other treatment methods has proven to reduce immunosuppressive processes, thereby augmenting the functional capacity of immune cells [216]. Some of these treatment methods include chemotherapy, radiation therapies, and the use of immune checkpoint inhibitors. Combination of DNA vaccines with chemotherapy has been shown to increase tumor antigen release, promotes T-cell activity, reduces immunosuppression, and has shown improved survival in preclinical models [217]. Additionally, in preclinical trials, vaccine and radiation therapies has enhanced tumor antigen release and cancer cell damage, and decreased tumor size [218]. Immune checkpoint inhibitors, such as CTLA-4 and PD-1 blockade augments antigen-specific T-cell activation, suppresses inhibitory cytokines including IL-10 and TGF-ß, and has demonstrated delayed tumor progression in preclinical and clinical studies [219,220].

DNA vaccines generally exhibit a favorable safety profile compared with alternative therapies as a result of their specificity and limited normal cell toxicity. Reported adverse events in early-phase clinical trials have primarily been mild (grade 1–2), such as injection site reactions or flu-like symptoms [203,205]. Grade 3 events have been uncommon and occurred in only a small number of participants. The safety profile of DNA vaccines in combination regimes varies depending on the partner therapy [216]. When combined with chemotherapy or radiation, side effects are typically attributable to the partnered treatment rather than the vaccine itself. The addition of immune checkpoint inhibitors may result in immune-related events, most of which are mild (grade 1–2) though severe events (grade 3–4) occasionally occur. Combining vaccine therapy with cytokine use may augment systemic reactogenicity, leading to more pronounced flu-like symptoms.

As research continues, these DNA vaccine therapies may offer new hope for patients by tailoring treatment to their unique tumor makeup and training their immune systems to respond more effectively.

## 5. Limitations in DNA Vaccine Effectiveness and Solutions

Several barriers may limit the therapeutic impact of DNA vaccines despite their successful clinical application. These challenges can be grouped into factors that impair the function of vaccine-induced T cells and barriers arising from the tumor microenvironment.

A hurdle for DNA vaccines includes immune tolerance, especially since tumor antigens utilized in DNA vaccines may be self-antigens typically bypassed by the immune system. A way to enhance the immunogenicity of DNA vaccines is to overcome immune tolerance. One method is to use xenogeneic version of antigens, which are homologous genes from another species to avoid tolerance and induce stronger immune responses. In a study where mice encoded human p53 gene, vaccination with a xenogeneic hp53 DNA construct delivered intramuscularly with electroporation generated a strong antibody response and promoted proliferation and activation of murine p53-specific CD8+ T cells. These CD8+ T cells were identified as the primary mediators of both prophylactic and therapeutic antitumor effects in murine colon cancer models. This demonstrates how xenogeneic antigens can overcome immune tolerance and enhance vaccine efficacy [221]. Another method involves encoding tumor antigens as fusion protein, such as linking them to immunoglobulin domains or forming single-chain trimers (SCTs) by utilizing MHC class I components. This method bypasses antigen-processing pathways by directly presenting peptides to CD8+ cytotoxic T cells while the IgG domain increases dendritic cell uptake and priming. In preclinical melanoma models, an SCT fused to tyrosinase related protein 2 (Trp2) enhanced CD8+ t cell responses and antitumor effects, demonstrating their potential to overcome tolerance to endogenous antigens [222]. These strategies allow for stable and direct antigen presentation to cytotoxic T cells.

Another hurdle is MHC class I downregulation by tumor cells, which allows tumors to evade CTL recognition. This reduction in MHC class I expression can result from a variety of mechanisms, including mutations, transcriptional silencing, epigenetic changes, disruptions in translation, and post-transcriptional or post-translational interference [223]. A key strategy to counteract this involves boosting MHC class I expression levels through treatments with pro-inflammatory cytokines, including IL-1, type I/II interferons, and tumor necrosis factor (TNF), or through the use of stimulator of interferon genes [224] or toll-like receptor (TLR) agonists [225]. Propper et al. showed that administering low doses of IFN-γ could elevate MHC class I and II levels in metastatic melanoma tissue [226]. Another approach involves the use of chemotherapy or radiotherapy to enhance antigen presentation, as these treatments can increase MHC class I expression. However, they may simultaneously promote an immunosuppressive environment, which could reduce overall treatment efficacy [227,228,229]. As a result, determining an optimal dosing strategy for such treatments remains an important area for further investigation. More recently, efforts have focused on small-molecule inhibitors aimed at restoring proper antigen presentation by targeting specific components of the regulatory pathways that suppress MHC expression [230]. However, identifying appropriate molecular targets would likely require genome-wide screening to uncover novel regulators involved in antigen processing and presentation [230].

An additional challenge that must be considered is the impact extrachromosomal DNA (ecDNA) has on the treatment process. ecDNA consists of large, circular DNA that can integrate into or separate from a chromosome, contributing to genomic remodeling through enhancer hijacking or disruption of tumor suppressor genes. They are often enriched in oncogenes and regulatory elements, and their circular structure leads to an open chromatin state that promotes high oncogene expression and novel gene-enhancer interactions [231,232,233]. Because ecDNA lacks centromeres, they segregate randomly during cell division, fueling tumor heterogeneity and accelerating clonal evolution. Their abundance can also change drastically under treatment pressure, with some ecDNAs expanding or disappearing to enable drug resistance [232,233]. Clinically, ecDNAs are now recognized as common across tumor types, and using data from the 100,000 Genomes Project, it is estimated that they can be found in approximately 17.1% of tumors, where their presence is associated with poor prognosis and unfavorable outcomes [231]. For DNA vaccines, this presents a significant problem. ecDNAs may alter antigen expression patterns and generate heterogeneity within the tumor, making it difficult to achieve effective immune control [234]. Conversely, their tumor-specific presence and role in shaping novel regulatory interactions may provide opportunities for vaccine targeting if ecDNA-derived neoantigens can be identified [235,236].

Another key challenge to the success of DNA vaccines and other immunotherapies is the immunosuppressive nature of the tumor microenvironment, which undergoes constant remodeling to support tumor progression [237]. This remodeling affects cancer cells as well as the surrounding blood vessels and stromal components such as cancer-associated fibroblasts, ultimately creating conditions that suppress immune cell activity [238,239]. One aspect of this suppression is the formation of physical barriers that limit immune cell infiltration. An example is the dense stromal architecture of PDAC that restricts T cell infiltration into and movement within the tumor tissue [240,241]. Several signaling pathways, including Hippo, TGFβ, and Wnt/β-catenin, have been implicated in the establishment of such barriers, and targeting these pathways has been explored to improve immune access [242,243,244]. Furthermore, impaired vasculature, commonly observed in tumors including PDAC, can reduce the efficiency of T cell trafficking into the tumor [245]. This insufficient vascularization is often accompanied by low levels of chemokines such as CCL4, CCL5, and CCL20, which are essential for recruiting dendritic cells to the tumor site for effective antigen presentation [246,247]. The use of chemotherapy or radiotherapy to enhance vessel permeability are ways to overcome these vascular limitations. In a notable study, Olive et al. demonstrated that inhibiting the hedgehog pathway could decrease stromal fibroblasts and improve blood vessel formation [248].

An additional limitation that must be taken into consideration is the variability of immune responses between individuals, which can be influenced by MHC class I diversity. Humans express a minimum of three and up to six HLA class I alleles, each with a distinct peptide-binding preferences. If the epitopes encoded by the vaccine, such as HPV E7-derived peptides, cannot be efficiently presented by a patient’s HLA molecules, then CD8+ T cell activation and subsequent antitumor immunity may be limited [234,249]. To address this, DNA vaccine constructs can be designed to include multiple epitopes from the same antigen or from several antigens, thereby increasing the likelihood of coverage across diverse HLA types [250]. Another approach is to focus on epitopes presented by common HLA alleles to maximize population-level effectiveness [251]. Importantly, personalized DNA vaccines tailor epitope selection to an individual’s HLA profile which offers a promising strategy to overcome this hurdle and optimize immune responses [252,253].

Beyond structural barriers, the tumor microenvironment contains immunosuppressive cells that interfere with T cell function. These immunosuppressive cells include fibroblasts associated with tumors, M2-polarized macrophages, regulatory T cells, and myeloid-derived suppressor cells [254,255]. One mechanism of T cell inhibition involves PD-1 on T cells interacting with its ligand PD-L1 present on tumor cells or macrophages in the tumor environment [256,257]. Additionally, immunosuppressive cytokines like IL-10 and TGFβ, and metabolic enzymes including indoleamine 2,3-dioxygenase (IDO), can hinder the activity and expansion of vaccine-induced T cells [258,259,260].

Another potential solution consists of adjusting the immune microenvironment to improve DNA vaccine potency. DNA plasmids co-administered with immunostimulatory molecules, such as IL-2, GM-CSF, or ligands for toll-like receptors (TLRs), can enhance dendritic cell activations and T cell priming [222]. CpG motifs in plasmid DNA can activate TLR9, while cytosolic DNA sensors can engage STING/TBK1 pathways leading to the production of type 1 interferons, which are crucial for effective immune responses [261]. Furthermore, stronger effector T cell responses can be accomplished by depleting regulatory T cells, allowing for unrestrained and enhanced immune activation [222].

On a broader scale, conditions such as acidic extracellular pH, hypoxia, and increased interstitial pressure within the tumor can suppress immune function [262]. Due to the evolving nature of the tumor microenvironment, addressing these immunosuppressive elements remains a significant obstacle. Current approaches include drugs that selectively target hypoxic regions and inhibitors that block immunosuppressive metabolites [263,264,265].

Elevated tumor burden and hypoxic conditions can contribute to T cell exhaustion, ultimately impairing the effectiveness of cancer vaccines [237]. Exhausted T cells show reduced functional capacity, diminished proliferation, elevated levels of inhibitory receptors, and an increased likelihood of cell death [266]. This dysfunctional state is primarily caused by prolonged exposure to tumor antigens, which leads to chronic TCR activation and subsequent metabolic stress, further exacerbated by the oxygen-deprived tumor microenvironment. To prevent or reverse exhaustion in vaccine-induced T cells, several strategies are being explored. These strategies include neoantigen vaccines combined with immune checkpoint inhibitors to restore T cell activity, targeting DNA methyltransferases such as DNMT1, DNMT3A, and DNMT3B that promote exhaustion-related epigenetic modifications, or attenuating hyperactive TCR signaling by inhibiting kinases that function downstream and are involved in chronic activation [267,268]. Additionally, modulating T cell metabolism, either by promoting the oxidation of fatty acids to favor memory T cell formation or by suppressing excessive glycolysis, may also enhance vaccine-induced responses. Another potential strategy involves combining vaccines with adoptive T cell transfer to increase therapeutic impact.

In cases of advanced or metastatic disease, neoantigen vaccines may be less effective on their own due to the larger tumor burden, heterogeneity of tumors, and evolving nature of metastatic lesions. As tumors spread, immune escape mechanisms such as epitope loss and clonal selection may arise, diminishing the ability of the immune system to identify vaccine-targeted antigens [269]. Moreover, distinct tumor microenvironments in metastatic sites can present further immunological barriers. In such scenarios, integrating neoantigen vaccines with other therapeutic approaches—including surgical resection, chemotherapy, radiotherapy, or additional immunotherapies—may be required to achieve more meaningful clinical outcomes [267].

## 6. Considerations in Clinical Trials

Personalized vaccines for cancer have been explored in two primary clinical contexts: (1) as adjunct therapy for early-stage, operable tumors and (2) as maintenance therapy for advanced or metastatic disease. In the adjuvant setting, vaccines are typically administered following surgical tumor removal. They are often combined with chemotherapy or immune checkpoint blockade to target any remaining cancer cells and prevent micrometastases from spreading. The main outcome measured in these clinical trials is usually recurrence-free survival (RFS). Early-stage trials commonly have larger patient populations but require longer follow-up periods, leading to higher overall costs and delaying outcomes. In contrast, when used for metastatic cancers, the focus is on stabilizing disease progression, with endpoints commonly including progression-free survival (PFS). Recruitment can typically be performed more quickly and yield results in a shorter time frame but may show limited benefit in late-stage patients whose immune systems are already weakened, constraining the therapeutic impact of immune-based interventions.

The encouraging outcomes observed in trials involving resectable cancers may be attributed to the ability of T cells to destroy residual cancer cells before the establishment of a highly immunosuppressive tumor microenvironment. In contrast, patients with late-stage malignancies often exhibit weakened immune responses, which can limit the effectiveness of therapeutic vaccination. As such, the interval between tumor biopsy and the actual administration of a personalized vaccine—commonly referred to as needle-to-needle time—is a critical factor in treating advanced cancers. One potential approach to bridge this gap is to begin treatment with a ready-made vaccine targeting shared tumor-associated or tumor-specific antigens (TAA/TSA), particularly for patients who express common HLA types. This allows for an immediate immune response while the patient-specific vaccine is still being manufactured, enabling a timely transition to personalized immunotherapy. Looking ahead, the administration of cancer vaccines in individuals with early-stage disease or pre-cancerous conditions could pave the way for a preventative strategy in high-risk groups. Such a prophylactic approach would involve the use of frequently observed neoantigens, identified through large-scale data mining across cohorts or public repositories including The Genotype-Tissue Expression (GTEx) project and The Cancer Genome Atlas (TCGA).

## 7. Conclusions

Multiple factors influence the efficacy of personalized DNA vaccines, such as the characteristics of selected neoantigens, the type of vaccine delivery system, the hurdles in vaccination, and whether the patient has an immunocompetent or immunocompromised immune system. The ideal neoantigens should be highly specific to tumor cells, largely clonal across cancer cell populations, and capable of triggering strong immune responses. While accurately predicting TCR binding and immunogenicity remains difficult, ongoing improvements in data quality and computational modeling continue to enhance prediction accuracy. Innovations in vaccine technologies have also contributed to progress in the field. However, it is important to recognize that different vaccine modalities may elicit distinct immune profiles, potentially favoring CD4+ or CD8+ T cell responses to varying degrees [270]. Additional challenges to address in future studies include overcoming the immune-suppressive tumor microenvironment, reversing T cell dysfunction, and managing the complexities posed by tumor burden and metastasis. Critically, a fully functional immune system is essential for translating vaccine-induced immune responses into therapeutic outcomes. Beyond activating T cells, vaccines must also promote the development of memory cells to maintain long-term tumor surveillance. To this end, many clinical trials are exploring DNA cancer vaccines in combination with checkpoint blockade therapies, conventional chemotherapy, or as adjuvant treatments following tumor resection in early-stage disease [271]. The latest advancements in vaccine platforms and bioinformatic tools have produced encouraging clinical results. As these technologies continue to evolve, DNA cancer vaccines are expected to become a key component of future immunotherapy strategies.

## Figures and Tables

**Table 1:** DNA Vaccines targeting Personalized Neoantigens.

Organ System	Vaccine Name	Vaccine Target	Target Disease	Delivery Method	Combination	Phase	Status	Outcome	NCT Number
Lymphatic System	scFv-CCL20 plasmid DNA vaccine	MIP3α-fused lymphoma idiotype	Lymphoplasmacytic lymphoma	DNA plasmid via I.D. injection with a needle-free bioinjector		I	Active, not recruiting	No results posted	NCT01209871 [203,204]
Breast	Personalized polyepitope DNA vaccine	4–20 patient specific neoantigens	TNBC	DNA plasmid via I.M. injection and electroporation	-	I	Complete	36-month recurrence-free survival: 87.5%	NCT02348320 [4]
Pancreas	Neoantigen DNA vaccine	Personalized neoantigen DNA vaccine	Pancreatic Cancer—surgically resected with adjuvant chemotherapy without evidence of recurrent disease	DNA plasmid via I.M. injection and electroporation	Chemotherapy	I	Terminated due to loss of funding	Treatment-related adverse events grade ≤ 3: 7/7	NCT03122106 [203]
Skin, Lung, Kidney, Bladder, Head and Neck, Breast, Cervix, Anus, Stomach/Esophagus, Colon	VB10.NEO	Up to 40 patient-specific neoantigens	Solid tumors	DNA plasmid via I.M. injection with a needle-free bioinjector	Atezolizumab—CPI (anti-PD-1 or anti-PD-L1), Bempegaldesleukin	I II/III	Complete	Stable disease: 34.8% (8/23) Neoantigen-specific response in stable disease patients: 100% (8/8)	NCT03548467 NCT05018273 [204]
Prostate	Neoantigen DNA vaccine	Patient-specific neoantigens	Prostate cancer	DNA plasmid via I.M. injection and electroporation	Nivolumab (anti-PD-1), Ipilimumab (anti-CTLA-4), PROSTVAC (PSA vaccine)	I	Complete	Treatment-related adverse events: Grade 3—13.3% (2/15) Grade ≤ 2: 86.7% (13/15)	NCT03532217 [205]
Liver	GNOS-PV02	Up to 40 patient-specific neoantigens	Hepatocellular carcinoma	DNA plasmid via I.D. injection and electroporation	INO-9012 and pembrolizumab	I/II	Active, not recruiting	Objective response: 30.6% (11/36) Complete response: 8.3% (3/36)	NCT04251117 [5]
Brain/Meninges	GNOS-PV01	Up to 30 antigens (27 TSA and 3 patient-specific)	Unmethylated glioblastoma	DNA plasmid via I.M. injection and electroporation	INO-9012 (plasmid encoding IL-12)	I	Active, not recruiting	No results posted	NCT04015700 [206]
	Personalized neoantigen DNA vaccine	Patient-specific neoantigens	Glioblastoma	DNA plasmid via I.M. injection and electroporation	Retifanlimab	I	Recruiting	No results posted	NCT05743595 [207]
	Personalized neoantigen DNA vaccine	Patient-specific neoantigens	Brain tumors	DNA plasmid via I.M. injection and electroporation	-	I	Recruiting	No results posted	NCT03988283 [208]
Kidney	Neoantigen DNA vaccine	Personalized neoantigen	Metastatic/advanced (inoperable) RCC	DNA plasmid via I.M. injection and electroporation	Durvalumab and tremelimumab	II	Withdrawn, FDA contingencies unresolved	No results posted	NCT03598816 [209]
Lungs	Neoantigen DNA vaccine	Patient-specific neoantigens	SCLC	DNA plasmid via I.M. injection and electroporation	Durvalumab, (and Carboplatin, Etoposide)	II	Active, not recruiting	No results posted	NCT04397003 [210]
Skin	EVX-02	13 neoantigens	Melanoma	I.M. injection	Nivolumab (anti-PD-1)	I/IIa	Terminated	No results posted	NCT04455503 [211]

## Data Availability

All data relevant to this review are included in the article. Data and materials are available upon reasonable request.

## Abbreviations

APC	Antigen-presenting cells
COVID-19	Coronavirus disease-2019
CPTAC	Clinical Proteomic Tumor Analysis Consortium
CTA	Cancer-testis antigen
CTL	Cytotoxic lymphocytes
DC	Dendritic cells
DNA	Deoxyribonucleic acid
EBV	Epstein–Barr virus
EGFR	Epidermal growth factor receptor
ELISpot	Enzyme-linked immunosorbent spot assay
FDA	Food and Drug Administration
FFPE	Formalin-fixed and paraffin-embedded
GM-CSF	Granulocyte macrophage-colony stimulating factor
GTEx	Genotype-Tissue Expression project
HBV	Hepatitis B virus
HCC	Hepatocellular carcinoma
HER	Human epidermal growth factor receptor
HLA	Human leukocyte antigen
HPV	Human papillomavirus
hTERT	Human telomerase reverse transcriptase
ICI	Immune checkpoint inhibitors
IDO	Indoleamine 2,3-dioxygenase
IFN-γ	Interferon gamma
IGFBP2	Insulin-like growth factor binding protein 2
IL	Interleukin
KRAS	Kirsten rat sarcoma virus
LNP	Lipid nanoparticles
MAGE	Melanoma antigen gene
MHC class I	Major histocompatibility complex class I
MHC class II	Major histocompatibility complex class II
mRNA	Messenger ribonucleic acid
NGS	Next-generation sequencing
NY-ESO-1	New York esophageal squamous cell carcinoma 1
PBMC	Peripheral blood mononuclear cells
PD-L1	Programmed death-ligand 1
PDAC	Pancreatic ductal adenocarcinoma
pMHC	peptide-MHC complex
PSA	Prostate-specific antigen
PSMA	Prostate-specific membrane antigen
RFS	Recurrence-free survival
RiboSeq	Ribosome profiling
RNA-seq	RNA sequencing
SNV	Single nucleotide variations
STING	Stimulator of interferon genes
TAA	Tumor-associated antigen
TCGA	The Cancer Genome Atlas
TCR	T cell receptor
TIL	Tumor infiltrating lymphocytes
TLR	Toll-like receptor
TMB	Tumor mutational burden
TNF	Tumor necrosis factor
TP53	Tumor protein p53
TSA	Tumor-specific antigen
WES	Whole exome sequencing
WGS	Whole genome sequencing
WT1	Wilms tumor 1
